# A Minimal Design of a Human Infant Presence: A Case Study Toward Interactive Doll Therapy for Older Adults With Dementia

**DOI:** 10.3389/frobt.2021.633378

**Published:** 2021-06-17

**Authors:** Hidenobu Sumioka, Nobuo Yamato, Masahiro Shiomi, Hiroshi Ishiguro

**Affiliations:** ^1^ Advanced Telecommunications Research Institute International, Kyoto, Japan; ^2^ Japan Advanced Institute of Science and Technology, Ishikawa, Japan; ^3^ Graduate School of Engineering Science, Osaka University, Osaka, Japan

**Keywords:** elderly dementia care, therapy robot, human–robot interaction, doll therapy, minimal design

## Abstract

We introduce a minimal design approach to manufacture an infant-like robot for interactive doll therapy that provides emotional interactions for older people with dementia. Our approach stimulates their imaginations and then facilitates positive engagement with the robot by just expressing the most basic elements of humanlike features. Based on this approach, we developed HIRO, a baby-sized robot with an abstract body representation and no facial features. The recorded voice of a real human infant emitted by robots enhances the robot’s human-likeness and facilitates positive interaction between older adults and the robot. Although we did not find any significant difference between HIRO and an infant-like robot with a smiling face, a field study showed that HIRO was accepted by older adults with dementia and facilitated positive interaction by stimulating their imagination. We also discuss the importance of a minimal design approach in elderly care during post–COVID-19 world.

## 1 Introduction

Dementia is a major cause of dependency and disability in older adults and significantly impacts them as well as their families, caregivers, and society. 40–50% of those with dementia suffer from cognitive, psychological, and behavioral problems (behavioral and psychological symptoms in dementia: BPSD), including hallucinations, depression, and agitation (Cerejeira et al., 2012). Such problems force caregivers to pay more attention to such people, which in turn increases their burden as well as the care costs. Therefore, BPSD reduction is a major social challenge. This is more urgent issue than ever due to the COVID-19 pandemic because recommended strategies such as social distancing taken during the pandemic inhibit daily activities and increase feelings of loneliness, which are potential factors that increase BPSD in older adults (Cohen-Mansfield et al., 2010). It also increases caregivers’ burden and financial costs for implementing proper measures to prevent infection.

Even though pharmacological interventions are often used to reduce problem behaviors, medical personnel generally recommend non-pharmacological interventions first to avoid side effects (Azermai et al., 2012). Next to face-to-face interaction with another person, a real baby, or a real animal, simulated social stimuli such as a lifelike (real) baby doll, stuffed animals, and robotic animals are the most engaging stimuli for older adults with dementia (Cohen-Mansfield et al., 2010). Doll therapy, which usually works by providing a human baby doll to older adults with dementia (Mitchell, 2016), is one type of non-pharmacological intervention with a simulated social stimulus. Studies report that older adults with dementia show the following care actions for the dolls: holding, talking, feeding, cuddling, or dressing, as well as increased levels of engagement with others and fewer problem behaviors (Mitchell et al., 2016). Although typical doll therapy uses a baby doll without any interactive functions, robotic technology can enhance the interaction with older adults. For example, Babyloid (Kanoh, 2015), which is a baby robot modeled on a baby beluga whale, is an application for interactive doll therapy because it can show reactions such as six kinds of emotional facial expressions as well as play a recorded voice of a one-year-old child and move its neck, mouth, and eyes in response to the actions of older people.

Since visual information of a doll is the primary factor in typical doll therapy, its appearance is the only factor that stimulates the imaginations of seniors with dementia and strengthens their feelings of human infant presence toward the doll. This is supported by the fact that they prefer a doll with a more humanlike appearance (Tamura et al., 2001). However, since a baby robot can interact with older adults in a multimodal way, both the interaction and the multimodal information from the robot are obviously important and must be designed carefully to stimulate their imaginations. One challenging technical approach is to make every feature as realistic as possible so that the robot actually resembles a human baby. However, due to the lack of technology, such an approach might cause an adaptation gap, which is why users’ final impression of artificial agents is negatively biased by the differences between the actual functions of artificial agents and those expected by them (Komatsu, 2011). Another potential problem is the uncanny valley effect, which describes the eeriness and discomfort when we encounter realistic virtual/artificial humans (Mori et al., 2012). Such approaches that design realistic, humanlike robot also increase both the robot’s expense and fragility, adding to the burden of care costs and fueling care staff resistance.

Another approach explores simpler designs to eliminate the negative effects described above. This approach simplifies the cognitive and socio-emotional information from a robot to increase user comprehension by streamlining it, and clarifies the information to be conveyed. In particular, this is helpful for older people because they have problems integrating information from multiple modalities due to cognitive and socio-emotional decline and mental illness (Ruffman et al., 2008; de Dieuleveult et al., 2017). In other words, conveying the information in fewer modalities probably helps older people understand the meaning of the information. This approach also helps us build a cost-effective and less-fragile robot. Therefore, an investigation of the minimal requirements of an interactive robot for doll therapy is critical to effectively achieve doll therapy.

In this study, we apply the minimal human design approach to an interactive baby robot to create a positive interaction for older adults with dementia by just expressing the minimum elements of humanlike features and stimulating the user’s imagination to supplement the missing information. We developed a tele-operated android named Telenoid as a test bed to investigate its effectiveness with older adults with dementia (Yamazaki et al., 2012; Kuwamura et al., 2016; Sumioka et al., 2014 for a review). Although Telenoid lacks functions to express facial emotions, older adults were able to imagine its smile from the information contained in the human operator’s voice. We expect that a design that facilitates user imaginations to a robot like Telenoid will enhance positive attitudes toward robots. Based on a minimal human design approach, we developed HIRO.^1^ Although its shape and size resemble a human baby, we eliminated facial and emotional expressions as well as detailed body parts such as hands. Instead of a simple appearance, we used the recorded voice of an actual human infant with multiple emotional states to strengthen a feeling of human infant presence and facilitate emotional interactions. HIRO vocalizes based on its emotional states, which respond to the actions of older adults as well as its own internal mechanism.

It remains unclear whether this new baby robot will actually be accepted by older adults with dementia and whether they will interact with it since it has an atypical appearance. This article investigates these points before performing interactive doll therapy, hypothesizing that a baby robot, which is designed based on the minimal human design approach, will induce longer interaction with dementia seniors than a baby robot with a face. We introduce HIRO and a baby robot with a face into an elderly nursing home to experimentally investigate how they influence older adults in their living environments. We evaluate whether seniors with dementia engage in positive, 5-min interactions with HIRO or a baby robot with a face. We also observe the interaction between older people and the robots, and identify their common and unique responses.

## 2 Related Work

Although a number of stimuli are used in non-pharmacological interventions, older adults with dementia prefer social stimuli (Cohen-Mansfield et al., 2010; Cohen-Mansfield et al., 2012). Cohen-Mansfield et al. compared 25 stimuli to determine which stimuli are most engaging, which are most often refused by dementia seniors, and which are most appropriate for persons who experience more difficulty engaging with stimuli (Cohen-Mansfield et al., 2010). They found that live social stimuli, including a real dog, real baby, and one-on-one interaction with another person, were the most engaging. Interestingly, a lifelike baby doll,^2^ which is an example of a simulated social stimulus, was also the most engaging stimuli. They also reported that seniors with low levels of cognitive functioning spent relatively more time responding to social stimuli than those with higher levels of cognitive functioning; women showed more attention and had more positive attitudes for simulated social stimuli than men. Therefore, the therapeutic use of a doll is a reasonable option as a simulated social stimulus for therapy.

Doll therapy has shown several potential therapeutic results (Bisiani and Angus, 2013; Mitchell and O’Donnell, 2013; Pezzati et al., 2014; Mitchell, 2016; Mitchell et al., 2016). A literature review on that approach by Mitchell et al. (2016) summarized four benefits: 1) it facilitates opportunities for communication with others; 2) it reduces distress including anxiety and problem behaviors, such as agitation and wandering; 3) it improves daily living activities, which include communicating, eating/drinking, and sleeping; and 4) it creates feelings of attachment, comfort, identity, and social inclusion. In doll therapy, a doll’s human-likeness seems important for enhancing its positive effects. A study on doll therapy reported that 13 out of 14 dementia seniors preferred dolls to stuffed bears (James et al., 2006). In addition, dolls with more humanlike features are preferred by Japanese seniors with dementia (Tamura et al., 2001). Therefore, an interactive baby robot may be more suitable for dementia sufferers to whom doll therapy is effective, although it remains unclear how the robot’s interactivity affects seniors with dementia.

Robot therapy has been proposed with several types of social robots to provide mental support for older adults. Some works suggest that effective interaction with a robot, such as touching and caring for older people, may improve their well-being by reducing anxiety, for example (Broekens et al., 2009; Valentí Soler et al., 2015; Pu et al., 2019). A seal-shaped companion robot, “PARO,” is one of the most famous robots for robotic therapy (Shibata, 2012; Takayanagi et al., 2014; Abbott et al., 2019). Many studies have shown that it provides not only psychological and physiological effects in older adults but also helps them expand their social networks (Wada and Shibata, 2007; Shibata, 2012). These effects have been confirmed in Japan and other countries (Shibata et al., 2009; Shibata and Coughlin, 2014). On the other hand, many seniors with dementia preferred dolls to stuffed animals in doll therapy (James et al., 2006). Therefore, an interactive baby robot may be more suitable for dementia sufferers to whom doll therapy is effective.

Babyloid (Kanoh, 2015), modeled on the beluga whale, is a baby robot that shows potential for interactive doll therapy. It can perform facial expressions with six types of emotions and multimodal reactions such as the recorded voice of a one-year-old and body movements. In a two-week experiment with five healthy seniors, their interaction with Babyloid averaged about 7 min, and their scores on the Geriatric Depression Scale (GDS), which measures depression, improved after the experiment. However, no studies have actually involved older adults with dementia. In addition, although various features have been implemented, no functional requirements have been explored.

We developed a tele-operated android named Telenoid based on a minimal human design approach to support communication with older people. Telenoid only has minimal functions for communication and a simple movement mechanism for its arms, neck, and mouth. It has no function for emotional expression, although it does have eyes, a nose, and mouth. Its appearance is gender neutral and ageless. However, the human operator’s voice information conveyed from Telenoid allows users to project personal traits and emotional states onto it. In fact, some elder adults with dementia claimed that Telenoid smiled during their interactions, although such a function was impossible (Sumioka et al., 2014). Another study described how older adults with dementia often made physical contact with Telenoids (Kuwamura et al., 2016). Such touch interactions probably increase the intimacy between older people and robots, and strengthen their social bonds (Okubo et al., 2018).

On the other hand, a human operator is required since Telenoid is a tele-operated robot. However, for seniors with moderate-to-severe dementia, verbal communication is often difficult, and simpler communication is probably sufficient. In addition, previous studies have shown that many such seniors treat Telenoid like a toddler even when adult care personnel tele-operated it (Yamazaki et al., 2012), suggesting that a more child-specific design that stimulates their imagination through simple communication may be sufficient. Therefore, we designed an interactive baby robot based on a minimal design approach.

## 3 Minimal Design of Human Infant

### 3.1 Visual Information

**FIGURE 1:**
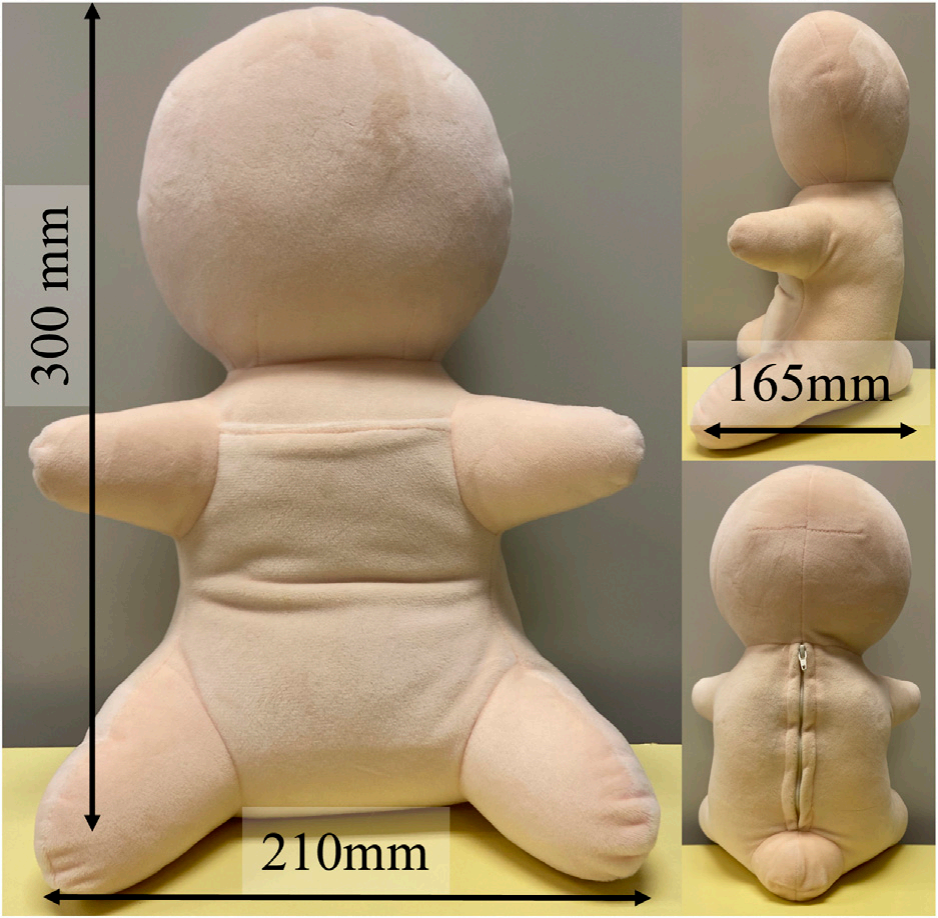
Minimal design of a human infant for interactive doll therapy, HIRO.

Figure 1 shows a prototype of our minimal design of a human infant, HIRO (W210×D165×H300 mm and 610 g). Its ABS control module, which is covered with a polyester fabric, includes a computer, a 3-axis accelerometer, and a speaker. The module can be removed from its back. Although HIRO is also equipped with a microphone and a touch sensor, we did not use them in this study since our purpose of this study was to explore minimal requirements.

Its appearance resembles that of a human baby. Distinctions among its head, torso, and limbs are recognizable, although it has no face. Previous studies confirmed that a distinction between the head and torso is the minimum requirement for enhancing the feeling of a human presence and that the expression of limbs does not significantly enhance human impressions (Sumioka et al., 2013). Although we could have removed the limbs, we left them so that older people can intuitively understand the robot’s orientation and its attitude to the interaction. The emotional mismatch between faces and voices complicates the perception of emotion (de Gelder and Vroomen, 2000), suggesting that if facial expressions are designed poorly, they may inhibit the transmission of emotions and negatively impact the human–robot interaction. For example, if the robot’s face expresses smiling but its voice expresses crying, the older adults may be confused about whether to continue the current action to maintain its smiling or take a different action to stop its crying. In addition, since older adults imagine facial expressions through communication with Telenoid (Sumioka et al., 2014), we excluded facial features to facilitate positive interactions.

### 3.2 Auditory Information

Instead of visual information, we enhanced human-likeness in the auditory information. We recorded the voice of a one-year-old infant and cut out 91 voice patterns. A male college student who was unaware of the purpose of this study categorized them into the following four emotions: positive: 20; weakly positive: 25; weakly negative: 17; and negative: 29. Positive and negative categories include voice patterns that are clearly either positive or negative. The voice patterns labeled relatively positive and negative were categorized into weakly positive and weakly negative. We also include three different babbling sounds, which denote meaningless articulations such as “ma ma ma,” in weakly positive. The rest of the sounds were various laughing and crying patterns. We expected that positive and weakly positive emotions facilitate the interaction between the robot and older adults with dementia. On the other hand, we were able to consider both positive and negative effects of negative and weakly negative emotions on the interaction with older adults with dementia: crying sound may induce caring behaviors from older people or it may also inhibit their caring behaviors.

### 3.3 Speech Generation Process

The design of the interaction between older adults and the robot is critical. The following is the robot’s speech generation process. The current internal state $s_{i}\left(t\right)$ is calculated with the previous state $s_{i}\left(t-1\right)$, the output value of the internal generator $s_{g}\left(t\right)$, and an external state that represents the effect of the interaction with older adults $s_{e}\left(t\right)$, which is reflected as sensor information from the 3-axis accelerometer. Based on the sum of these three values, $s_{i}\left(t\right)$ is selected among negative 1), weak negative 2), weak positive 3), and positive 4), and a current voice is randomly chosen from the selected emotional category. For example, if $s_{i}\left(t\right)=4$, one of the voices classified as positive is randomly selected. In this study, the internal generator generated +1, 0, and −1 with a probability of 50, 20, and 30%, respectively, which suggests that the robot tended to express positive emotions. The external state $s_{e}\left(t\right)$ was changed based on the L2 norm of three values of the accelerometer ($ACC_{L2}$) so that it reacts when the robot is lifted up or set down. If the L2 norm exceeds a certain threshold ($Th_{ACC}$), we set $s_{e}\left(t\right)=1$; otherwise, $s_{e}\left(t\right)=0$. This implies that the robot’s emotional state is more often positive when there is more interaction with the participant and less often positive when there is less interaction. For example, HIRO often laughs when a user lifts or rocks it. On the other hand, it often cries when a user places it on her/his legs or just talks to it without lifting or rocking it. If $s_{i}\left(t\right)$ is higher (lower) than 4 (1), we set $s_{i}\left(t\right)=4$ ($s_{i}\left(t\right)=1$). This processing interval was randomly changed within 2–5 s. In this study, we set the initial state to $s_{i}\left(0\right)=1$, which means HIRO has a negative emotional state and often cries at the beginning of the interaction.

## 4 Experiment

HIRO is an interactive doll robot that uses minimal visual information and humanlike emotions transmitted by voice to stimulate the imagination of dementia patients and induce positive interaction with them. Since this is the first attempt to introduce a faceless robot into elderly care, we should first address the following three research questions (RQs) before applying HIRO to practical doll therapy:

*RQ1 Will a robot based on the minimal design of a human infant (HIRO) actually be accepted by olders?*

We applied a minimal design approach because we expected it to stimulate the imagination of dementia seniors and encourage emotional interaction between HIRO and seniors as in previous studies with Telenoid. Therefore, we also investigated the following question:

*RQ2 If HIRO is accepted, will it be more positively accepted than a baby robot, which has more body representations and facial expressions?*

Since HIRO has no facial features, which are important for face-to-face interaction, perhaps the type of the interaction between HIRO and seniors with dementia will be different from that between a robot, which has facial features, and such seniors. Therefore, we addressed the following question:

*RQ3 Does HIRO elicit different interaction patterns from a baby robot which has facial features?*

### 4.1 Participants

Our experiment was carried out in an elderly nursing home in Hyogo Prefecture with 21 older (18 women) participants. Their average age was 86.6 years (SD: 5.4). Their average level of required long-term care (care level) was 3.38 (2–5), based on Japanese government guidelines, which is determined by how much care the person requires.^3^ For example, a senior with care level 2 needs partial assistance with daily activities such as personal hygiene concerns and bathing, and shows some confusion and decreased understanding. A senior with care level 5 needs complete care for every aspect of his or her life and shows many anxiety behaviors and a general decline in understanding. Seniors were selected by the nursing home’s personnel to join the experiment from the seniors who actively participate in the events held in the nursing home and those who had difficulty speaking but care staff members wanted to observe the interaction with the robot. All the participants and their families received information and signed informed consent forms approved by the ATR Ethics Committee and with their doctor’s permission.

### 4.2 Procedure

**FIGURE 2:**
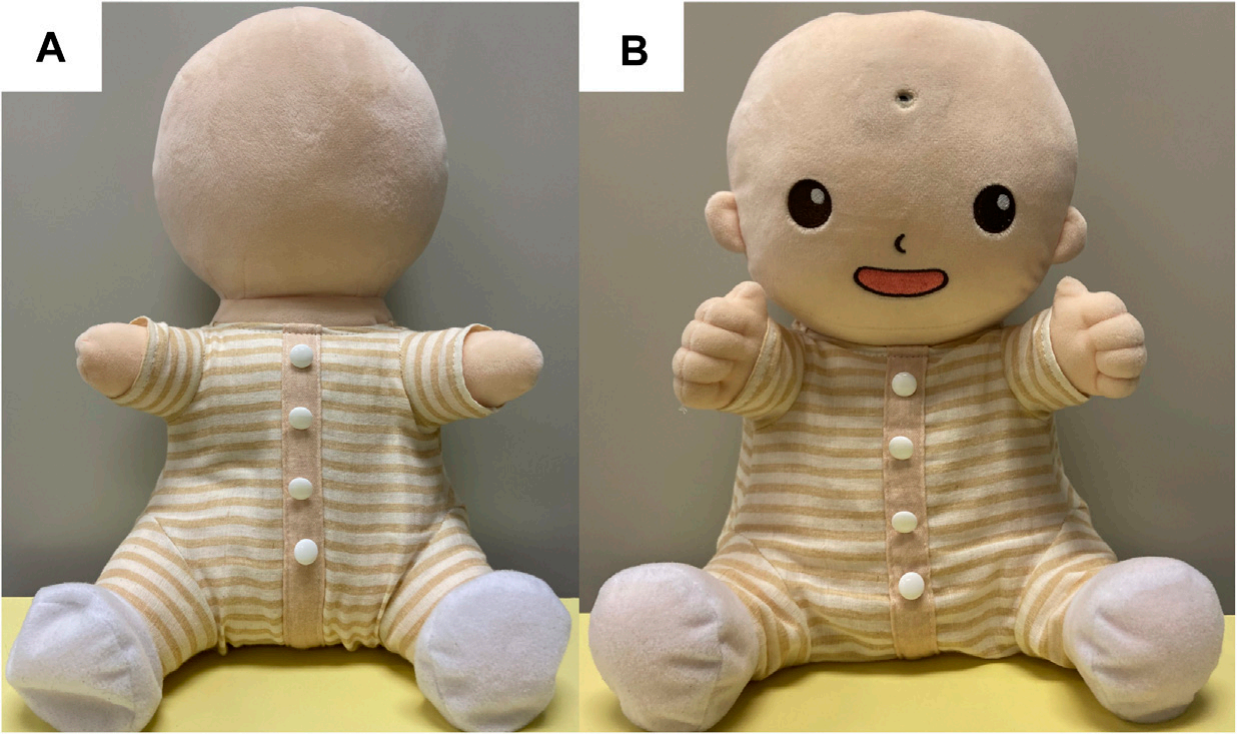
**(A)** HIRO in the no-face group and **(B)** a baby robot with a face in the face group.

Participants were randomly divided into two groups: participants in one group (11 participants, two males) were given a HIRO (no-face group), and those in the other group (10 participants, one male) were given a robot with a more humanlike appearance (a robot with a face hereafter), as shown in Figure 2 (face group). This robot has a smiling face, ears, and fingers. It also has a small camera inside its head. This was implemented for a purpose of telecommunication, although we did not used it. Both robots wore identical clothing (Figure 2). All participants were parents who had raised more than one child, except for one participant in each group. Two participants who have speech difficulty were in the no-face group and one was in the face group. One bedridden participant also joined each group. The demographic information of the participants in each group is shown in Table 1. In this table, F, M, and O denote the participant IDs that is used in the Result section.

**TABLE 1:** Demographic information of participants. F, M, and O indicate the participant IDs.

Group	Age (SD)	Care level (SD)	Sp^a^	Be^b^
No-face	86.3(4.21)	3.36(0.81)	M, O	M
Face	86.9(6.62)	3.4(0.97)	F	F

a Participants with speech difficulty.

b Bedridden participants.

We investigated whether the participants would continue to hold their baby robot and positively interact with it for 5 min after taking it from a staff member with whom they are familiar. This 5-min interval was suggested by the care staff members themselves because it represents a minimum interval that can reduce their work burdens. The experiment was carried out in the participants’ private rooms. Those who had no problem walking sat in chairs in their room. People in wheelchairs sat in them. Bedridden people remained in their beds.

The following is our experiment’s procedure. The staff and an experimenter entered the participant’s room, introduced the robot they were holding, and gave it to the participant to hold and soothe (pre-interaction phase). The staff and experimenter treated the robot like a human baby. This phase helped the participants understand the robot’s function and how they should deal with it. The experimenter set a video camera in the room, began video recording, and left.

About a minute later, the staff member got a call from the experimenter and explained to the participant: “*He (the experimenter) needs my help. Do you mind looking after at this baby?*” The staff member exited the room, leaving the participant and the robot alone (interaction phase). In this phase, there was a possibility that the request by the staff member forces the participant to continue to care for the robot. To avoid this possibility, we asked the care staff member to stay in the room with half of participants in each group. We expected that the participants who did not want to care for the robot to pass it to the staff member without any hesitation. In this case, the care staff avoided actively interacting with them. During the interaction phase, the experimenter (and the staff member if they had left from the participant’s room) observed the situation in the room from outside by an external monitor.

We observed the interaction between the senior and the baby until the 5-min interval was satisfied or until any of the following scenarios occurred: the participant lost interest in the robot, such as dropping it or falling asleep; and the participant summoned the staff member if she was not present or passed the robot to the staff member if she was present. Next, the experimenter (and the staff member if she had left) returned to the room, thanked the participant for caring for the robot, retrieved both it and the video camera, and left the room. After the experiment, we interviewed the staff members to get their reactions to the participant interactions with the robot, especially for any opinions about how the states/actions of their patients had changed. We also collected the histories of the robot’s emotional states in the interaction phase.

### 4.3 Evaluation

We investigated RQ1 by examining whether the older participants accepted this robot for the entire 5 min. We identified those who continued to hold HIRO or the robot with a face for 5 min as those who accepted it, and those who stopped holding it or returned it to the staff member in less than 5 min as those who refused the robot. We counted the participants who accepted the robot and calculated the percentages. We performed the one-sample proportion test to assess whether the percentage in each group was higher than the chance level. We also compared the percentages in two groups using the chi-squared test.

Regarding RQ2 and RQ3, we analyzed the care behavior of the participants to investigate their attitude toward the robot in the interaction phase. We first determined behaviors such as caressing, hugging, rocking, or talking to the robot, singing, and other visible behaviors during interaction with a human infant as caring behaviors. We also determined the behavior of just holding the robot without talking to it as no-caring behaviors. A coder who was unaware of the purpose of the study encoded the behaviors and the duration of each one in all the recorded video/audio data. Then another coder who was also unaware of the purpose of the study coded 10% of these data. We calculated the coding’s validity based on the previous work (Lombard et al., 2002). The kappa coefficient was 0.79, indicating substantial agreement between the two coders. In this study, we did not encode the facial expressions of the participants because some of their faces were hidden by the robot and correctly identifying their facial expressions was difficult. If the participants showed at least one caring behavior toward the robot, we judged that they showed a positive attitude toward it. We investigated RQ2 by evaluating the difference of the duration of the caring behaviors between the two groups. We used Welch’s t-test since the normality was confirmed with the Shapiro–Wilk test.

To investigate RQ3, we also analyzed the proportion of emotional states selected by the robot during the interaction and examined how their frequency affected the interaction times with older adults. We evaluated the difference of the proportion of the robot’s emotional states between the two groups. We used Welch’s t-test when we confirmed normality with the Shapiro–Wilk test, while Mann–Whitney’s U test was used when normality was not confirmed. We also conducted a correlation analysis for each emotional state of the robot and the duration of the caring behaviors of the participants to investigate whether the participant’s behaviors are correlated with the robot’s emotional states. If the participants respond to the robot’s behavior (e.g., laughing), their behavior should increase as the ratio of the robot’s emotional state (e.g., positive state) increases. We used Pearson’s correlation because we predicted the robot’s emotional states have a linear relationship with the duration of the participant’s caring behaviors.

In addition to these quantitative evaluations, we observed the participants’ speech and behavior toward the robot to investigate RQ3 and extracted the shared and different characteristics of the participant responses. We also interviewed three care staff members who supported our experiments and compared their impressions to the more typical reactions of the participants.

## 5 Result

### 5.1 Quantitative Results

Regardless of the group, most participants accepted the robot. 60% in the face group and 81.8% in the no-face group continued to hold it for the entire 5 min. One sample proportion test showed that the no-face group was significantly higher than a chance level (50%) ($\chi^{2}=4.45,p=.017$) but the face group was not ($\chi^{2}=.4,p=.26$). Six participants rejected the robot in total. The average duration of rejection was 161.4 (SD:51.5) seconds. In the face group, four participants refused the robot: one participant immediately stopped holding it after she was left alone, and three participants returned the robot to the staff member after less than 5 min. In the no-face group, two participants rejected the robot. One participant threw it on her bed, and another fell asleep while caring for it. Although more participants continued to hold the robot in the no-face group, we found no statistically significant difference in terms of proportions using the chi-squared test ($\chi^{2}=1.22,p=.27,95\text{\%}CI=\left[-0.16,0.60\right]$).

**FIGURE 3:**
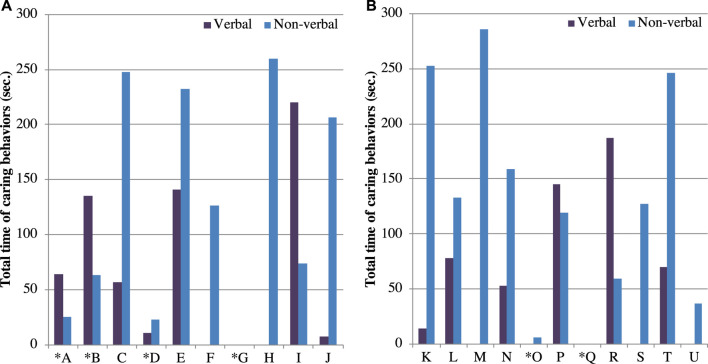
Total time of caring behaviors presented by each participant during interaction in the **(A)** face group and **(B)** no-face group. Letters indicate each participant. * shows participants who refused the robot. The alphabets denote the participant IDs.

We also analyzed the caring behaviors of the participants to investigate their attitudes toward the robot and how HIRO affected their interaction with the seniors. Almost all participants showed some caring behaviors to the robot, which indicate that they showed positive attitudes toward it. All the participants who accepted the robot showed positive attitudes toward it. Figure 3 shows the total duration of the caring behaviors exhibited by each participant. The alphabets in this figure denote the participant IDs. We separately show the caring behaviors from two aspects: a verbal aspect, which consists of talking to the robot and singing; and a nonverbal one, which includes caressing the robot, hugging and rocking it, and other behaviors that are commonly seen during interaction with a human infant. An asterisk indicates participants who refused the robot. Even the participants who refused it showed a few caring behaviors. We extracted the participants who both accepted the robot and showed positive attitudes toward it from each group (i.e., nine participants in the no-face group and six participants in the face group). The average duration of verbal caring behaviors was 70.8 (SD:91.1) seconds in the face group and 60.8 (SD:67.8) seconds in the no-face group; the average duration of the nonverbal caring behaviors was 191.0(SD:74.6) seconds in the face group and 157.7(SD:87.1) seconds in the no-face group. We evaluated the differences in the duration of the verbal caring behaviors and nonverbal caring behaviors between the two groups. We applied Welch’s t test for both behaviors since the normality was confirmed with the Shapiro–Wilk test. We found no significant difference between groups in the verbal caring behaviors (t=.23,p=.82,d=.13) or in the nonverbal caring behaviors (t=.79,p=.44,d=.41).

**FIGURE 4:**
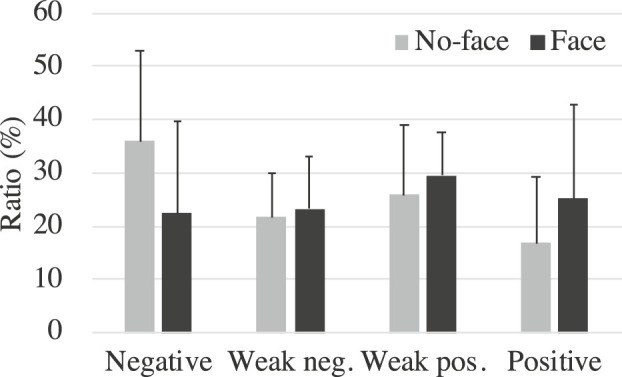
Average ratio of robot’s emotional states during interaction.

**FIGURE 5:**
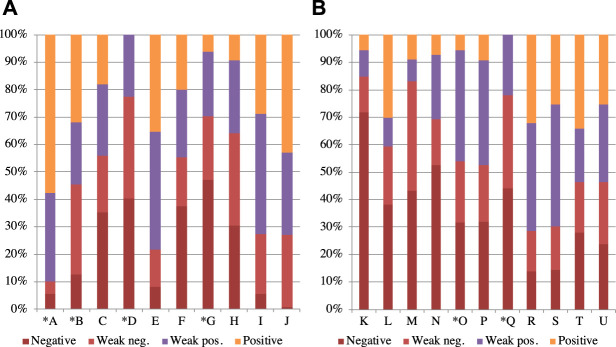
Ratio of robot’s emotional state during interaction in the **(A)** face group and the **(B)** no-face group. Letters indicate each participant. * shows participants who refused the robot. The alphabets denote the participant IDs.

We also investigated the proportion of the robot’s emotional states that occurred during the interaction to determine whether the participants engaged differently with the robot across the conditions. Figure 4 shows the average of the ratio of the emotional states of the robots over a 5-min period for each group. Since the normality in the negative and positive states was confirmed with the Shapiro–Wilk test but not in the weak negative and weak positive ones, we did Welch’s t-test and Mann–Whitney’s U test for the weak negative and weak positive states. Although the no-face group tended to have more negative states than the face group (t(18.7)=−1.80,p=.089,d=.79), there were no significant differences (weak negative: W=63,p=.60,r=.17; weak positive: W=68,p=.39,r=.28; positive: t(16.1)=1.23,p=.24,d=.55). Figure 5 shows the percentage of the emotional states that occurred during the interaction with each participant. The alphabets in this figure denote the participant IDs. An asterisk indicates participants who rejected the robot for the entire 5 min. We found no distinctive differences between the groups or whether they interacted with it for the entire 5 min.

Finally, we conducted a correlation analysis for the ratio of each emotional state of the robot and the total time of the verbal/nonverbal caring behaviors of the participants to investigate any potential relationship between them. Since the sample size of each group is small, the analysis was conducted for all participants who accepted the robot in both groups. Pearson’s correlation showed two significant correlations in the verbal caring behavior (Table 2). We found a negative moderate correlation between verbal behavior and the robot’s negative state (r=−0.47,p=.03). This indicates that the more often the robot expressed negative emotion such as crying, the less often the participants talked to it. We identified a positive moderate correlation between verbal behavior and the robot’s weak positive state (r=0.44,p=0.046), which means that when the more often the robot expressed a weak positive state, the more often the participants talked to it. We could not find any significant correlation in other relationships, although we found a trend that approached significance for a positive correlation between verbal behavior and the robot’s positive state (r=0.40,p=0.070).

**TABLE 2:** Correlation coefficients between caring behavior and the robot’s state.

Caring	Robot’s internal state			
Behavior	Negative	Weak neg	Weak pos	Positive
Verbal	−0.47*	−0.28	0.44*	0.40^†^
Nonverbal	0.15	0.01	−0.32	0.05

### 5.2 Qualitative Results

#### 5.2.1 Observation Results

The participants who interacted with HIRO or the robot with face for 5 min showed positive attitudes toward them. They treated the robot as a typical actual infant; they caressed the robots, sang to them, lifted them up, kissed, cuddled, and rocked them. Even in the no-face group, participants interacted while looking at the robot’s face, even though it had no facial expression. The participants who were able to communicate addressed it regardless of the condition: “*It’s so cute*” or “*What’s your name?*” They talked to it about things related to the robot and themselves.

The participants also responded to the robot’s crying and laughing: “*Don’t cry*” or “*It’s laughing*.” They tried to interpret the robot’s babbling. For example, participant R said, “*Don’t say no*” in response to the robot’s babbling, and participant L said, “*It said that its name is Kentaro*.” A senior with severe dementia (participant O) who had difficulty communicating parroted the robot’s voice.

In the no-face group, participants L and T talked about HIRO’s face. Participant T, looking at HIRO, remarked that “*he has no eyes or mouth!*” Participant L also lamented that he had no eyes or mouth while pointing at his face. However, the lack of facial expressions did not interfere with their interactions because they both continued to hold HIRO and showed positive attitudes toward it.

Many of the participants actively cared for the robot even though they knew that it was a robot. For example, participant T said, “*It’s sturdy.*” Participant E asked, “*What’s the doll’s name?*” On the other hand, two participants seemed confused about whether the robot was actually human. For example, when HIRO started crying, in the no-face group, participant N, who has severe dementia, said, “*I can’t breastfeed him*,” which implies that she thought HIRO was hungry. Participant C who has severe dementia in the face group repeatedly touched the robot’s crotch and tried to undress it when it started crying. The staff member suggested that “*it looked like she was checking the robot’s diaper.*”

#### 5.2.2 Interview With Care Staff Members

All three of the staff members pointed out that the participants smiled when they interacted with both the robots. For example, one staff member mentioned the change in facial expression of subject C. He said, “*Her expression changed from before she interacted with the robot. She was smiling during the interaction*.” Another staff member mentioned that the interaction with the robot made some participants feel more awake. He said about subject C, who was a night person and slept a lot during the day, “*She was fully awake. I think she’ll sleep well at night.*” On the other hand, the other staff member mentioned that the participants who usually showed aggressive behavior were calm. She said, “*I was surprised that Subject S, who usually wanders around and behaves aggressively, was quiet and still*.”

They also pointed out the effect of baby voices. One staff member said, “*I think many participants accepted the robot because of the baby’s voice*.” Another staff member said, “*I think that a baby’ voice from the robot stimulated the seniors since they usually just hear the voices of us staff members and rarely hear babies*.”

The care staff members agreed that the participants were willing to care for HIRO, although they seemed to realize that it was a doll. One staff member also pointed out the clarity of their utterances: ”*I think that participants tried to speak more clearly than usual because they thought they were talking to a baby*.”

## 6 Discussion

### 6.1 Implication

We introduced a minimal design approach and developed an interactive doll called HIRO to investigate the necessary elements for a robot for interactive doll therapy. HIRO had minimal representation as a human baby and no facial features. Nevertheless, the older participants continued to hold it and manifested positive attitudes toward it. Therefore, we can answer yes to RQ1: the older adults accepted HIRO, even though it had a very simple representation of a human infant. We also confirmed that they showed a positive attitude toward HIRO, suggesting that it has potential to provide dementia sufferers with active and affective engagement, which plays a protective role against cognitive decline and reduces problem behaviors (Cohen-Mansfield et al., 2010).

We developed RQ2 and RQ3 expecting that eliminating nonessential information and allowing users to imagine the missing information would promote positive interaction and that the lack of facial features would encourage different interactions with dementia seniors. Unfortunately, we found no significant effect compared to the robot with face, which means that the answer to RQ2 is no. The participants’ observations confirmed that both robots elicited a variety of imaginative reactions from older adults, which indicate that the answer for RQ3 is also no. Since our experiment was only 5 min long, perhaps its impact may not have been significant. We need to test longer and multiple interactions.

Another possibility is that the lack of information about appearance may not have had a great effect because Japanese people tend to rely more on auditory information than European people when visual and auditory information are presented simultaneously (Tanaka et al., 2010). Maybe interaction through human voices by robots has become integral for older people with dementia. This result also suggests that simple appearance of HIRO may be less effective with Europeans than the Japanese because they tend to reply more on visual information. Although no cultural differences were found in previous studies with Telenoid, perhaps its humanlike appearance will work more effectively with Europeans, and its human voice is effective with the Japanese. We look forward to investigating cultural differences in robot design guidelines in interactive doll therapy.

### 6.2 Effects of Voice Characteristics

The human baby voice strongly affected the seniors in our study. It also seemed to affect the behaviors of the seniors since the care staff felt they tried to speak more clearly, as described in Section 5.2.2. In this study, we used a recorded voice of an actual human infant. However, it remains unclear how important the human-likeness of a voice is for facilitating the interaction between a robot and older people. It will be interesting to examine whether a similar effect is achieved in the future when low-quality speech or synthetic speech are used.

However, a human baby voice is not always acceptable for older adults. Some participants became upset or rejected the robot when it started crying. Strongly negative emotions from the robot seemed to upset the participants. This reaction is also supported by a negative correlation between verbal caring behaviors and the ratio of the robot’s negative states, which showed an increase in the robot’s negative state and a decrease of the verbal caring behaviors. This result suggests that a robot should basically avoid expressing strongly negative emotions because older adults might feel stress about handling the crying robot. In contrast, a few participants, especially women, enjoyed handling the crying robot. Therefore, it should have a function to control the frequency of the expression of strongly negative emotions based on the characteristics of older users. It might also be interesting to characterize the relationship between the ratio of emotional expressions and the personalities of older user.

### 6.3 Effect of Forming Attachment With a Baby-Sized Robot on Older Adults With Dementia

When people are relieved of concerns with regard to instrumental goals for the future, their attention is directed to their emotional states, and as a result, emotionally fulfilling interactions with family and friends become important for them (Van Assche et al., 2013). However, they have increasingly experienced loss of loved ones. Moreover, age-related diseases such as dementia present a further challenge for many aging individuals. Studies report that the experience of loss is associated with dementia and BPSD (Van Assche et al., 2013). Several interventions such as reminiscence therapy, animal therapy, robot therapy, and doll therapy focus on utilizing dementia patients’ own attachments and have been shown to be effective in preventing BPSD. Particularly, animal therapy, robot therapy, and doll therapy help dementia patients develop new attachment relationships with animals, robots, and dolls. A study with PARO reports that dementia patients show greater interest in PARO than in a static, stuffed toy (Takayanagi et al., 2014). Therefore, we expect that HIRO not only develops the attachment relationship with dementia patients but also strengthens it since it shows emotional responses, such as crying and laughing, to them, compared with a static doll. It is interesting to compare HIRO with a static doll with respect to the development of attachment relationship as a future work.

### 6.4 Effect of Minimal Design on Caregivers and an Elderly Nursing Home

Multimodal interaction is important for rich emotional interaction, and humanoid robots are an effective means to do so. On the other hand, increasing the quality of every modality increases the cost and fragility of robots. The minimal design approach, which searches for the minimum necessary elements to facilitate emotional interaction, limits the robot’s functions, resulting in a cheaper and more robust machine. In fact, HIRO-chan, the commercially available version of HIRO, is less expensive (about $49) than other robots and interactive toys, which usually cost more than $1,000.^4^ Since it is covered with a soft material, it is reasonably safe from being damaged even when it is handled carelessly or roughly. These are big advantages compared to other robots for robotic therapy, especially during the COVID-19 pandemic when it is difficult to share a robot with others, and the burden on care staff has become a serious problem. Due to the low-cost design of a robot, the managers of elderly facilities and family members of dementia sufferers can provide this robot to every senior who requires it. Care staff members can also use it with less concern about damage because of its robustness. Therefore, we argue that a minimal design is an important approach to apply robots to the new lifestyle realities that are surfacing due to the COVID-19 pandemic.

Our results also suggest that perhaps care staff can take their eyes off seniors with dementia and do other tasks for brief periods by asking seniors with dementia to care for HIRO. This provides opportunities for them to spend time on other activities, which decreases their work burden. A staff member who did not support our experiment but observed it mentioned other potential effects of a baby robot on caregivers: “*When I heard the robot’s laughter during my work, I felt good. That laughter might improve the atmosphere in the nursing home and reduce such serious problem as older adult abuse by caregivers since it makes them relaxed.*” We will investigate the effect of HIRO on the mental and physical stress of caregivers in the future work.

### 6.5 Potential Social Effect

In animal therapy and robot therapy, it is well known that animals or robots facilitate communication between older people and other people. We also observed this possibility in this experiment. When the care staff member was present in the participant’s room, the participant often talked to the staff member about the robot. Since we requested the member to avoid actively interacting with the participant in this study, HIRO did not facilitate communication between the staff member and the participant. However, if the care staff members can communicate freely, they could facilitate communication with the older adults using the baby robot. We also observed this tendency in the case of the participants who were left alone. Some participants actively talked to both the care staff member and the robot when the staff member gave it to them. However, they did not interact much with the robot once the staff member left the room. Therefore, it is interesting to verify the robot’s effectiveness in a group situation where caregivers or other senior people are present.

### 6.6 Difference From Another Minimally Designed Robot

We found various aspects between the baby robot and another robot developed with a minimal design approach, the Telenoid. Telenoid is a tele-operated android robot, and its purpose is to facilitate verbal communication between its tele-operator and older people. Therefore, it is challenging to continue interacting with older people with dementia who have difficulty speaking. On the other hand, HIRO does not assume verbal communication, and therefore, even such people can continue to interact with it. For example, Subjects F and M enjoyed the interaction with HIRO even though they had difficulty speaking. It also seems that older people with dementia guess different ages between Telenoid and HIRO. A previous study with Telenoid reported that dementia people confused Telenoid with a five-year-old child (Yamazaki et al., 2012). On the other hand, as the care staff pointed out, they considered HIRO as a baby and tried to speak more clearly than usual. Such modulation of adult speech to HIRO is similar to adult speech to human babies known as “motherese,” which means that adults’ language to babies is simpler than the language spoken among adults (Snow, 1977). Therefore, it would be an interesting issue to examine whether HIRO induces more motherese from senior people than Telenoid.

### 6.7 Ethical Consideration

Although doll therapy has been shown to improve the well-being of older people with dementia, some researchers point out the ethical issues of this technique from a concept of infantilization, which is defined as treating a person in a patronizing way or as a parent might treat their very young child (Tim, 1997), and ethical discussions have taken place (Mitchell and Templeton, 2014). Mitchell and Templeton suggest that doll therapy has the potential to be truly person-centered care, which is underpinned by respect, understanding, and self-determination to dementia patients, if it is utilized in a meaningful way, such as giving the dementia patients a choice to use them, rather than forcing them to use them (Mitchell and Templeton, 2014). Although a doll therapy with an interactive robot should share some ethical issues with a typical doll therapy, it is necessary to establish guidelines for caregivers to utilize the robot appropriately, referring to the guidelines for typical doll therapy. The Convention on the Rights of Persons with Disabilities (CRPD) recommends research and development of universal design that can meet the needs of persons with disabilities with minimum possible adoption and the least cost. Interactive doll therapy with HIRO may be viewed as one such low-cost, easily accessible means to meet needs of older adults with dementia for social interaction as well as enhance their well-being.

### 6.8 Limitations

There are several limitations in this study. First, its sample size was relatively small and the degree of dementia was not balanced across the groups. We characterized the participants by governmental care levels, which is a domestic measure and includes difficulty in both physical and mental aspects. However, the acceptance of a simulated social stimulus and the duration of engagement with it depend on the level of the senior’s cognitive function (Cohen-Mansfield et al., 2010). Therefore, future experiments with larger samples must separately measure the participants’ dementia levels and their daily activities with objective indicators such as Severe Mini Mental State Examination (sMMSE) and Physical Self-Maintenance Scale (PSMS). We must also objectively assess whether HIRO actually reduces problem behaviors. Due to such large individual differences, future research must perform validation in a within-subject design.

The significant gender bias of the participants may also affect our results since there were a few male participants in this study. It is well known that nearly two-thirds of people with dementia are women (Fukawa, 2018). In fact, in the nursing home where we conducted the experiment, male residents only accounted for about 17% of all residents (22 persons). Previous studies also report their results with very small male participants (Cohen-Mansfield et al., 2010; Pezzati et al., 2014; Kuwamura et al., 2016). It is necessary to conduct experiments in several nursing homes to increase male participants to verify the influence on male older adults with dementia.

Although this study showed no significant difference between a baby-sized robot with no facial features and one with facial features, it remains unclear whether a more humanlike appearance enhances the interaction between older adults with dementia and a baby robot. It is attractive to compare HIRO with a baby-sized robot with a much more humanlike appearance to investigate how a humanlike appearance facilitates the interaction with older people with dementia. Comparisons with other social interaction–focused therapies, such as a doll therapy and robot therapy with animal robots such as PARO, are also important to characterize this therapy.

We judged acceptance of HIRO by 5-min interaction between HIRO and older people with dementia. The experiment’s duration is relatively short, compared with other human–robot interaction studies, although it is comparable with other psychological studies (Cohen-Mansfield et al., 2010; Pezzati et al., 2014). We selected 5 min as a minimum interval to reduce the work burdens of the care staff members. As a next step, we need further research on how long the senior people with dementia will engage with the robot and how many times they will engage with it to verify the robot’s effectiveness and reduce caregiver’s work burden.

## 7 Conclusion

We introduced a minimal design approach to manufacture a robot for interactive doll therapy. Based on this approach, we developed HIRO, a baby-sized robot with an abstract body representation and no facial features. Emotional interaction was achieved with the voice of an actual baby to activate the imagination of seniors with dementia. Fieldwork for seniors with dementia showed that they accepted HIRO. Additional experiments with more samples are needed in the future. We also have to verify whether it actually reduces behavior problems using objective indicators. Social isolation due to the COVID-19 pandemic is causing serious problems in elderly care facilities in terms of the mental health of both the patients and their caregivers. We hope HIRO helps to not only improve their mental health but also function as a novel cost-effective tool in robot therapy that does not need to be shared.

## Data Availability

The raw data supporting the conclusions of this article will be made available by the authors, without undue reservation.

## Footnotes

1 HIRO-chan is a commercial version manufactured by Vstone Co., Ltd. https://www.hiro-chan.jp/en/.

2
https://en.wikipedia.org/wiki/Reborn_doll.

3
https://www.mhlw.go.jp/english/topics/elderly/care/index.html.

4 There are several cheap baby robots that were developed for older adults. For example, Kabochan Nodding Communication Robot (PIP Co., Ltd., Osaka, Japan and WiZ Co., Ltd., Tokyo, Japan) costs about $231. Smiby, which is a commercial version of Babyloid (TOGO SEISAKUSYO CORPORATION, http://www.togoh.co.jp/products/care-smiby.html), costs about $627.
